# Efficacy and Safety of Chemotherapy or EGFR‐TKIs as First‐Line Therapy in NSCLC Patients Harboring Non‐Ex 20 Ins Uncommon EGFR Mutations: A Retrospective Study in China

**DOI:** 10.1002/cam4.70542

**Published:** 2024-12-30

**Authors:** Chen Liao, Li Bai, Tingting He, Qingle Liang, Defeng Hu, Shipeng Lei, Yong He, Yubo Wang

**Affiliations:** ^1^ Department of Respiratory and Critical Care Medicine Chongqing University Jiangjin Hospital Chongqing China; ^2^ Department of Respiratory and Critical Care Medicine, Xinqiao Hospital Army Medical University Chongqing China; ^3^ Department of Respiratory and Critical Care Medicine, Daping Hospital Army Medical University Chongqing China; ^4^ Department of Clinical Laboratory Medicine Chongqing University Jiangjin Hospital Chongqing China

**Keywords:** efficacy, EGFR‐TKI, NSCLC, safety, uncommon EGFR mutation

## Abstract

**Background:**

Uncommon EGFR mutations are a kind of heterogeneous group of mutations with various responses to EGFR‐TKIs and are often excluded from most prospective clinical trials. In this real‐world retrospective study, we retrospectively compared the efficacy and safety of chemotherapy or various generations of EGFR‐TKIs as first‐line therapy in NSCLC Chinese patients harboring non‐ex 20 ins uncommon EGFR mutations.

**Methods:**

We enrolled 139 NSCLC patients with non‐ex 20 ins uncommon EGFR mutations in this study retrospectively. Patients' clinical characteristics and the efficacy and safety of different first‐line therapies were analyzed and compared.

**Results:**

Our data reviewed that for first‐line therapy, NSCLC patients harboring non‐ex 20 ins uncommon EGFR mutations benefited more from EGFR‐TKIs compared with chemotherapy. Afatinib performed with great efficacy for the majority of non‐ex 20 ins uncommon EGFR mutations (*N* = 43, ORR = 41.86%, mPFS = 13.5 months, mOS = 20.8 months), especially in L861Q mutation (mPFS = 18.4 months). Osimertinib also demonstrated efficacy in patients harboring non‐ex 20 ins uncommon EGFR mutations (*N* = 36, ORR = 27.78%, mPFS = 10.0 months, mOS = 21.0 months), especially in those without L861Q and G719X mutations (mPFS = 12.1 months). When treated with afatinib, patients harboring non‐ex 20 ins uncommon EGFR mutations should pay attention to the management of safety, especially for gastrointestinal‐related AE and rash, while osimertinib was safer.

**Conclusion:**

Taking into account both efficacy and safety, afatinib and osimertinib are better choices than chemotherapy and first‐generation EGFR‐TKIs for NSCLC patients with non‐ex 20 ins uncommon EGFR mutations. L861Q showed a trend toward a better response to afatinib, while in those without L861Q and G719X mutations, osimertinib might be a better choice. Safety also should be a concern when choosing EGFR‐TKI for treatment, patients should pay attention to the management of safety when using afatinib while osimertinib is safer.

## 1 Introduction

Non‐small‐cell lung cancer (NSCLC) accounts for about 85% of all lung cancers and is one of the leading causes of death related to cancer in China [1]. With the identification of oncogenic driver mutation and the progression of targeted therapy, the survival of NSCLC patients has been greatly prolonged. Epidermal growth factor receptor (EGFR) mutation is the most common oncogenic driver mutation in NSCLC and can be found in about 10%–15% of Caucasians and around 50% of Asian patients with NSCLC [2]. EGFR common mutation or classical mutation which accounts for about 90% of all EGFR mutations includes EGFR exon 19 deletion and EGFR exon 21 L858R mutation. EGFR common mutation can benefit from EGFR tyrosine kinase inhibitor (EGFR‐TKI) treatment [3, 4, 5]. Thus, EGFR‐TKIs including first‐generation TKIs [6, 7, 8], second‐generation TKIs [4, 9], and third‐generation TKIs [10, 11, 12] have been used as the first‐line treatment of patients with common EGFR mutation. However, data on the efficacy treatment of uncommon EGFR mutation which accounts for 10% of EGFR mutations is still insufficient and need more exploration.

Uncommon EGFR mutations are kind of heterogeneous mutations with various responses to EGFR‐TKIs and are often excluded from most prospective clinical trials [13, 14]. In a small‐scale prospective study, osimertinib showed 50% ORR with median progression‐free survival (mPFS) of 8.2 months in 37 advanced NSCLC patients with uncommon EGFR mutations [15]. Dacomitinib also demonstrated efficacy in 32 NSCLC patients harboring uncommon EGFR mutations with an ORR of 56.3% and mPFS of 10.3 months [16]. Pooled analysis of LUX‐Lung 2, LUX‐Lung 3, and LUX‐Lung 6 clinical trials suggested that afatinib is effective in NSCLC tumors with uncommon EGFR mutation [17]. However, few researchers have compared the efficacy of various EGFR‐TKIs and chemotherapy in NSCLC patients with uncommon EGFR mutations. More explorations are needed to evaluate the better EGFR‐TKI options considering mutation specificity.

Considering that EGFR‐TKIs have little efficacy in patients harboring EGFR 20 insertion (20 ins) mutation, in our present study, 139 NSCLC patients harboring uncommon EGFR mutation excluding EGFR 20 ins mutation were enrolled. Their clinical characteristics and sensitivity to different EGFR‐TKIs or chemotherapy were analyzed.

## 2 Methods

### 2.1 Patients and Data Collection

**FIGURE 1:**
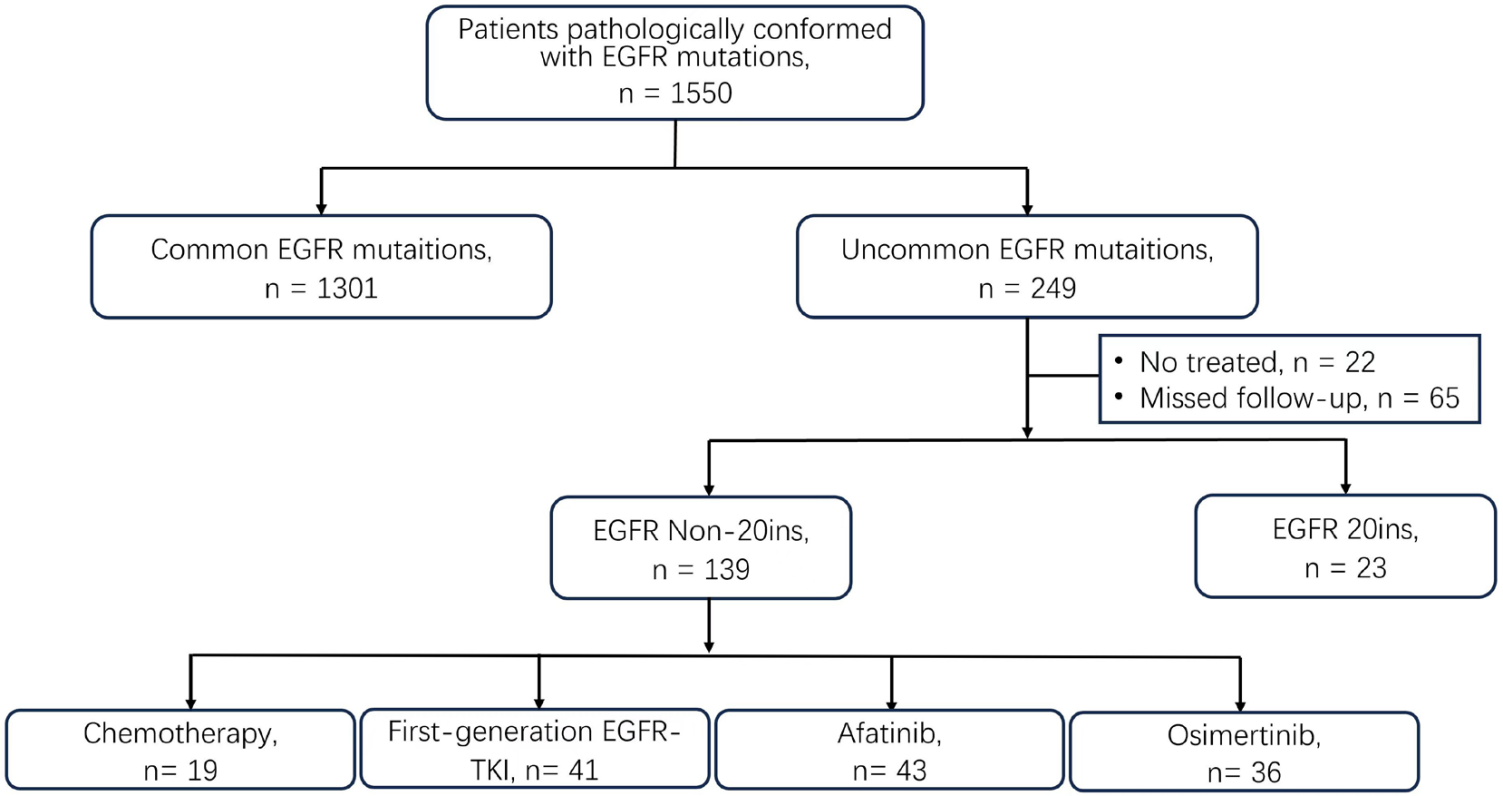
A flowchart of the patient cohort. EGFR‐TKI, epidermal growth factor receptor tyrosine kinase inhibitor; NSCLC, non‐small cell lung cancer; first‐generation drugs, gefitinib, erlotinib, and icotinib were included.

As seen in Figure 1, in this retrospective study, patients diagnosed with advanced NSCLC harboring uncommon EGFR mutation excluding EGFR 20 ins mutation in three hospitals (Daping Hospital, Chongqing University Jiangjin Hospital, and Xinqiao Hospital) from 2016 to 2022 were enrolled. Finally, 139 patients were enrolled and followed up until June 2023 or death.

The clinical characteristics and demographic data such as sex, age, smoking history, baseline EGFR mutation, and adverse events (AEs) of patients were collected. AEs were graded according to the Common Terminology Criteria for Adverse Events (CTCAE) version 4. Genetic status was detected by ARMS PCR or NGS technology. Patients with only one EGFR mutation were classified as simple EGFR mutation, while patients with more than one EGFR mutation were defined as complex EGFR mutation. This study has been approved by the Ethics Committee of Chongqing University Jiangjin Hospital [Ratification No.: KY2023024] and the informed consent waiver has been obtained. We guarantee that this study was conducted in accordance with the Declaration of Helsinki.

### 2.2 Treatment and Response Evaluation

Patients with non‐ex 20 ins uncommon EGFR mutation were treated with chemotherapy or different EGFR‐TKIs such as first‐generation (gefitinib, erlotinib, and icotinib), second‐generation (afatinib), or third generation (osimertinib) EGFR‐TKIs in the first line until progression or intolerable toxicity. Tumor response was evaluated by computed tomography according to Response Evaluation Criteria in Solid Tumors 1.1 criteria every 2–3 months. The definition of PFS was the time from the start of EGFR‐TKI or chemotherapy treatment until disease progression or death. The definition of overall survival (OS) was the time from the start of EGFR‐TKI or chemotherapy treatment until death or last follow‐up.

### 2.3 Statistical Analysis

The chi‐square test was performed to evaluate the differences in clinicopathological features between different groups. The Kaplan‐Meier method and log‐rank test were performed to estimate and compare the PFS and OS, respectively. All statistical analyses were performed by IBM SPSS Statistics for Windows (version 23.0). A *p*‐value less than 0.05 was considered statistically significant.

## 3 Results

### 3.1 Patients' Characteristics

In 1550 NSCLC patients pathologically conformed with EGFR mutations, 195 (16.1%) patients with uncommon EGFR mutation were found. Excluded untreated patients or patients who missed follow‐up and patients with EGFR 20 ins mutation, a total of 139 advanced NSCLC patients with non‐ex 20 ins uncommon EGFR mutation were enrolled in this study and divided into four groups according to their first‐line therapy: first‐generation EGFR‐TKI group (*n* = 41), afatinib group (*n* = 43), osimertinib group (*n* = 36), and chemotherapy group (*n* = 19). Patients' characteristics are shown in Table 1. The demographic data for these 139 patients revealed that women occupied a higher proportion than men (55.4% vs. 44.6%), while non‐smokers occupied a higher proportion than smokers (58.3% vs. 41.7%). Most patients were stage IV (89.2%, 124/139), and 9.4% (13/139) patients had central nervous metastasis. Among these 139 patients, 72 patients had complex mutations, including 18 patients with uncommon and common mutations and 54 patients with uncommon and uncommon mutations. G719X and its complex mutation account for 33.8% (47/139), L861Q and its complex mutation accounts for 26.6% (37/139), and S768I only accounts for 4.3% and other uncommon EGFR mutation accounts for 35.3% (49/139), respectively.

**TABLE 1:** Patients' characteristics.

	First‐generation EGFR‐TKI *N* = 41 (%)	Afatinib *N* = 43 (%)	Osimertinib *N* = 36 (%)	Chemotherapy *N* = 19 (%)	*p*
Age (years)	59.95 ± 9.97	61.27 ± 9.98	66.63 ± 10.38	58 ± 11.09	0.009
Gender					
Male	18 (43.9)	18 (41.9)	11 (30.6)	15 (78.9)	0.007
Female	23 (56.1)	25 (58.1)	25 (69.4)	4 (21.1)	
Smoking history					
Yes	18 (43.9)	17 (39.5)	10 (27.8)	13 (68.4)	0.036
No	23 (56.1)	26 (60.5)	26 (72.2)	6 (31.6)	
Pathological type					
Adenocarcinoma	38 (92.7)	42 (97.7)	32 (88.9)	18 (94.7)	0.246
Squamous carcinoma	1 (2.4)	0	0	1 (5.3)	
Others	2 (4.9)	1 (2.3)	4 (11.1)	0	
Stage					
IIIB–IIIC	4 (9.8)	7 (16.3)	2 (5.6)	2 (10.5)	0.528
IV	37 (90.2)	36 (83.7)	34 (94.4)	17 (89.5)	
Central nervous metastasis					
Yes	2 (4.9)	3 (7.0)	5 (13.9)	3 (15.8)	0.252
No	39 (95.1)	40 (93.0)	31 (86.1)	16 (84.2)	
EGFR sensitizing mutation					
G719X and its complex mutation	15 (36.6)	20 (46.5)	8 (22.2)	4 (21.1)	0.0001
L861Q and its complex mutation	9 (22.0)	15 (34.9)	10 (27.8)	3 (15.8)	
S768I	3 (7.3)	1 (2.3)	2 (5.6)	0	
Others	14 (34.1)	7 (16.3)	16 (44.4)	12 (63.1)	

Then, we compared baseline characteristics among these 4 groups. Patients in the osimertinib group were much older (66.63 ± 10.38 years) and had more female patients than the other groups (*p* = 0.009 and *p* = 0.007, respectively). The Osimertinib group had more non‐smokers while the chemotherapy group had much fewer non‐smokers (*p* = 0.036). The distribution of various EGFR mutations among these 4 groups also showed some difference: in the afatinib group, nearly half of the patients had G719X and its complex mutation (46.5%, 20/43), while in the chemotherapy group, 63.1% (12/19) patients had other uncommon EGFR mutations (*p* = 0.0001). Moreover, no significant difference in pathological type (*p* = 0.246), stage (*p* = 0.528), and central nervous metastasis rate (*p* = 0.252) were identified among these 4 groups. The multivariable analysis indicated that age (*p* = 0.078), gender (*p* = 0.956), and smoking history (*p* = 0.910) were not the independent predictive factors associated with PFS.

### 3.2 Treatment Outcomes

**FIGURE 2:**
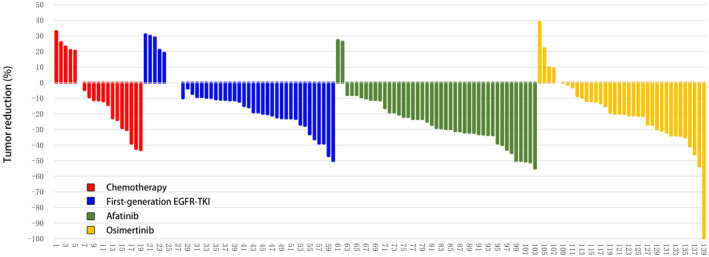
ORRs of patients with uncommon EGFR mutations receiving different EGFR‐TKIs or chemotherapy as first‐line therapy.

For these 139 patients with non‐ex 20 ins uncommon EGFR mutations receiving different EGFR‐TKIs or chemotherapy as first‐line therapy, we further analyzed the efficacy of different therapies. As shown in Figure 2, the ORR of afatinib was 41.86%, which was significantly superior to other therapies (ORR range 14.63%–27.78%). The DCR of afatinib (95.35%) and osimertinib (97.22%) were similar and were both superior to other therapies (first‐generation EGFR‐TKI: 87.80% and chemotherapy: 73.68%).

**FIGURE 3:**
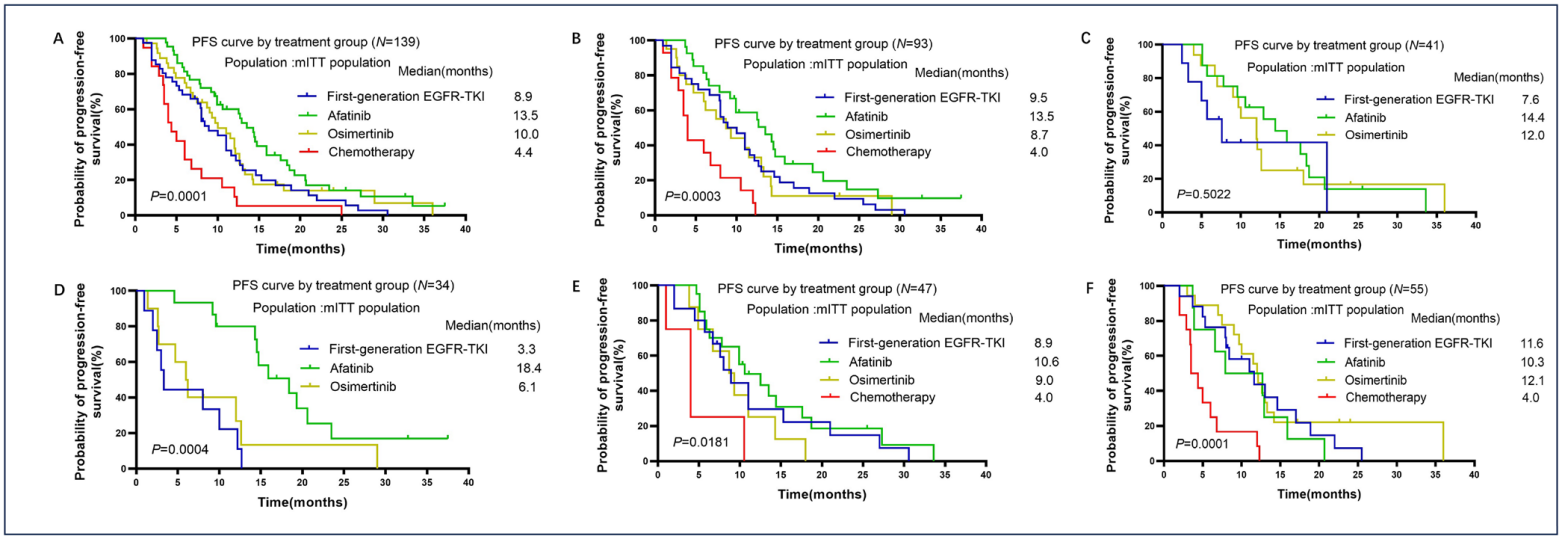
Kaplan–Meier curves of median PFS among patients with (A) all types of uncommon EGFR mutations in different groups, (B, C) simple and complex uncommon EGFR mutations, (D) L861Q EGFR mutation, (E) G719X EGFR mutation, and (F) other uncommon EGFR mutations except L861Q and G719X.

As shown in Figure 3A, compared with chemotherapy as first‐line (mPFS = 4.4 months), all EGFR‐TKI treatment groups were associated with much longer mPFS, of which the afatinib group showed the longest mPFS (mPFS = 13.5 months, *p* = 0.0001). Then we analyzed mPFS of different groups of patients with simple EGFR mutation or complex EGFR mutation, respectively. Our results demonstrated that in patients harboring simple EGFR mutation, afatinib was markedly superior to other EGFR‐TKIs and chemotherapy in mPFS (Figure 3B, mPFS 13.5 months vs. range 4.0–8.7 months, *p* = 0.0003). In the patients harboring complex EGFR mutation, afatinib also showed longer mPFS than other EGFR‐TKI groups although the difference was not significant (Figure 3C, mPFS 14.4 vs. range 7.6–12.0 months, *p* = 0.5022). Considering the heterogeneity of uncommon EGFR mutations, we further analyzed mPFS for patients with L861Q, G719X, and other uncommon EGFR mutations of different treatment groups. Our data indicated that in patients harboring L861Q and its complex mutation, afatinib presented remarkably longer mPFS than other treatments (Figure 3D, mPFS 18.4 vs. range 3.3–6.1 months, *p* = 0.0004). In the patients harboring G719X and its complex mutation or other EGFR uncommon mutations, all EGFR‐TKIs groups demonstrated significantly longer mPFS than the chemotherapy group (Figure 3E,F, *p* = 0.0181 and *p* = 0.0001, respectively). But mPFS in different EGFR‐TKI groups were similar in these two subgroups, although afatinib showed longer mPFS in patients with G719X and its complex mutation (mPFS = 10.6 vs. range 8.9–9.0 months) and osimertinib seemed to reach a longer mPFS numerically in patients with other uncommon EGFR mutations (mPFS 12.1 vs. 10.3–11.6 months). We also observed that patients treated with first‐line chemotherapy had the shortest mPFS which was about 4 months among every subgroup.

**FIGURE 4:**
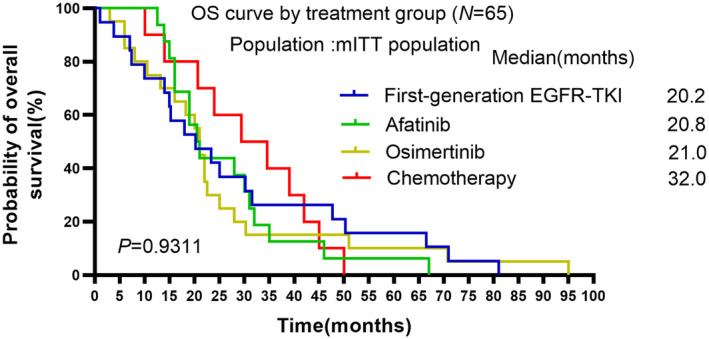
Kaplan–Meier curves of median OS among patients with uncommon EGFR mutations receiving different EGFR‐TKIs or chemotherapy.

Finally, OS was compared among these four groups. Nevertheless, OS was not significantly different among these groups (Figure 4, mOS: 20.2 in the first‐generation group, 20.8 in the afatinib group, 21.0 in the osimertinib group, 32.0 in the chemotherapy group, *p* = 0.9311).

### 3.3 Adverse Events

**FIGURE 5:**
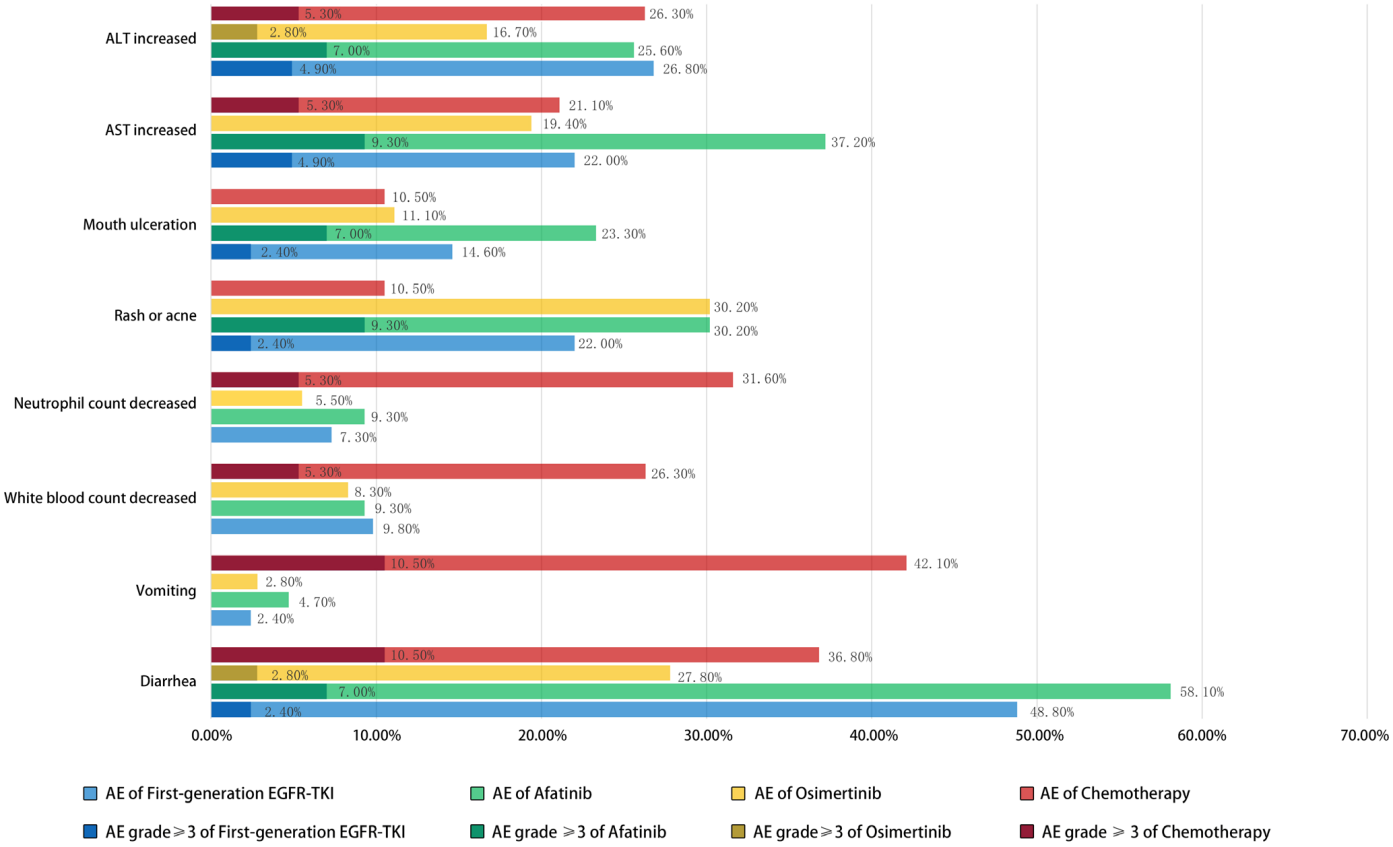
Occurrence rates of common AEs of all grades and ≥ 3 grade among different groups.

Common AEs were recorded and summarized in Table 2 and Figure 5. Patients treated with afatinib or chemotherapy experienced more gastrointestinal‐related AE‐like diarrhea (all stages 58.1% and 36.8%, respectively) and vomiting (all stages 4.7% and 42.1%, respectively) than those treated with first‐generation EGFR‐TKI or osimertinib. Patients in the chemotherapy group experienced more white blood count decreased (all stage 26.3%) and neutrophil count decreased AEs (all stage 31.6%) than other groups. As for rash or acne and mouth ulceration AEs, the rates of the afatinib group were much higher than other groups (all stages 30.2% and 23.3%, respectively). The ALT and AST increased AEs occurred similarly among the first‐generation, afatinib, and chemotherapy groups, but much lower in the osimertinib group. Patients in the osimertinib group seemed to experience fewer AEs compared with other groups.

**TABLE 2:** Details of AEs in different groups.

	First‐generation EGFR‐TKI	Afatinib	Osimertinib	Chemotherapy	*X* ^2^	*p*
	*N* = 41 (%)	*N* = 43 (%)	*N* = 36 (%)	*N* = 19 (%)		
Diarrhea	12 (48.8)	25 (58.1)	10 (27.8)	7 (36.8)	10.209	0.017
≥ 3 grade	1 (2.4)	3 (7.0)	1 (2.8)	2 (10.5)	2.545	0.420
Vomiting	1 (2.4)	2 (4.7)	1 (2.8)	9 (42.1)	23.783	0.000
≥ 3 grade	0	0	0	2 (10.5)	6.239	0.018
White blood count decreased	4 (9.8)	4 (9.3)	3 (8.3)	5 (26.3)	4.069	0.247
≥ 3 grade	0	0	0	1 (5.3)	4.223	0.137
Neutrophil count decreased	3 (7.3)	4 (9.3)	2 (5.5)	6 (31.6)	7.806	0.038
≥ 3 grade	0	0	0	1 (5.3)	4.223	0.137
Rash or acne	9 (22.0)	13 (30.2)	3 (8.3)	2 (10.5)	6.898	0.072
≥ 3 grade	1 (2.4)	4 (9.3)	0	0	4.254	0.141
Mouth ulceration	6 (14.6)	10 (23.3)	4 (11.1)	2 (10.5)	2.493	0.477
≥ 3 grade	1 (2.4)	3 (7.0)	0	0	2.914	0.341
AST increased	9 (22.0)	12 (37.2)	7 (19.4)	4 (21.1)	0.897	0.847
≥ 3 grade	2 (4.9)	4 (9.3)	0	1 (5.3)	3.510	0.288

## 4 Discussion

In the present study, the efficacies of different EGFR‐TKIs or chemotherapy as first‐line therapy for NSCLC patients harboring non‐ex 20 ins uncommon EGFR mutations were retrospectively investigated. Although the FDA had approved afatinib for the treatment of NSCLC patients with uncommon EGFR mutations, this use did not been approved in China. Therefore, there were still some patients with uncommon EGFR mutations who selected chemotherapy or other EGFR‐TKIs as their first‐line treatment. Our study highlighted that all generations of EGFR‐TKI showed better efficacy than chemotherapy for patients with non‐ex 20 ins uncommon EGFR mutations. Taking into account both efficacy and safety, afatinib and osimertinib are better choices than chemotherapy and first‐generation EGFR‐TKIs for NSCLC patients with non‐ex 20 ins uncommon EGFR mutations. To the best of our knowledge, this real‐world study is the first study to compare the efficacy and safety of three generations of EGFR‐TKI and chemotherapy in NSCLC patients harboring non‐ex 20 ins uncommon EGFR mutations.

In accordance with previous studies, women, non‐smokers, and adenocarcinoma made up a higher proportion of all the patients with uncommon EGFR mutation [18]. Considering that EGFR‐TKIs have little efficacy in patients harboring EGFR 20 ins and there are already drugs approved by the FDA for EGFR 20 ins, we excluded these types of patients from our present study. In line with the published manuscript, except EGFR 20 ins, G719X and L861Q mutations were the most frequent [19, 20, 21].

The heterogeneity of uncommon EGFR mutation made variable sensitivity to different EGFR‐TKIs [22, 23, 24, 25]. We observed that compared with chemotherapy, receiving any generations of EGFR‐TKIs in the first line is a better choice for patients with uncommon EGFR mutations, for the reason that mPFS of chemotherapy was only about 4 months in our study and was the shortest one among the groups. Xu's research also revealed that patients with uncommon EGFR mutations who received EGFR‐TKIs experienced a significantly prolonged OS than those who did not (mOS 18.96 vs. 12.22 months, *p* = 0.017). Moreover, consistent with other studies, afatinib demonstrated better efficacy in most uncommon EGFR mutations [17, 26, 27]. Yang's [17] study which was based on a pooled analysis of the LUX‐Lung 2, LUX‐Lung 3, and LUX‐Lung 6 clinical trialss reported that afatinib was effective in 75 NSCLC patients harboring uncommon EGFR mutations, with an ORR of 41.3%, a mPFS of 10.7 months and a mOS of 19.4 months. In another clinical trial of 315 NSCLC patients with uncommon EGFR mutations received afatinib treatment, reaching an mPFS of 10.8 months [28]. The first phase III clinical trial to compare efficacy and safety of afatinib versus chemotherapy for the treatment of NSCLC patients with uncommon EGFR mutations indicated that patients in the afatinib group had significantly longer mPFS compared with patients in the chemotherapy group with similar ≥ 3 grade AEs incidence rates in both groups (mPFS 10.6 vs. 5.7 months, *p* = 0.0007; ORR 61.4% vs. 47.1%, *p* = 0.2069) [28]. We obtained similar results with an ORR of 41.86%, mPFS of 13.5 months, and mOS of 20.8 months in the patients with uncommon EGFR mutations treated with afatinib, validating the efficacy of afatinib. Especially for patients with L861Q mutation, afatinib might be the better choice as the first‐line treatment, for it can reach an mPFS of 18.4 months in our study. This finding was consistent with some preclinical studies indicating that L861Q mutation responds better to afatinib than first‐generation EGFR‐TKIs according to their IC_90_ value [29, 30]. The ORR in the osimertinib group is lower than other studies (ORR around 50%–60%), but the DCR (around 90%), mPFS (around 9–10 months), and mOS (around 21–25 months) were similar among our study and other studies [31, 32]. The PS score, types of EGFR uncommon mutations, metastasis organs, and so on can all influence the efficacy data of treatment. Maybe this is the reason that the ORR in our study is different from other studies.

In this present study, we observed that the survival of patients with complex mutations appeared to be better than patients with simple uncommon EGFR mutations. Previous research found that patients with complex uncommon mutation had significantly better ORR and mPFS than those with single uncommon mutation (ORR 68.4% vs. 37.8%, *p* = 0.011; mPFS 11.9 vs. 6.5 months; *p* = 0.010) [33]. Passaro's [34] study also reported similar outcomes in that patients with complex mutations had longer PFS and OS than those without (mPFS: 12.9 vs. 7.9 months; mOS: 35.1 vs. 15.0 months). Complex uncommon EGFR mutation might co‐exist with classical EGFR mutation and thus might be more sensitive to EGFR‐TKIs than those with simple uncommon EGFR mutation. However, there needs more crystal structure analysis to elucidate the mechanisms of these observations. Shen's [35] study reported that afatinib showed longer mPFS than gefitinib/erlotinib in NSCLC patients harboring non‐classical mutations (mPFS 11.0 vs. 3.6 months, *p* = 0.03). The PFS curves of afatinib were more easily distinguished in non‐classical EGFR mutations lacking a combination with a classical mutation. Maybe afatinib is a good choice for patients with simple uncommon EGFR mutation.

Numerous of researches have compared the efficacy of afatinib with first‐generation EGFR‐TKIs, but few compared the efficacy of afatinib with osimertinib in NSCLC patients with uncommon EGFR mutations. Two studies from Qin et al. and Si et al. [18, 24] enrolled patients with uncommon EGFR mutations treated with osimertinib, but the sample size was too small with one and four patients in each study, respectively. A PubMed database‐based literature review and pooled analysis from Wang found that both afatinib and osimertinib showed favorable clinical activities in treating uncommon EGFR mutations. Afatinib displayed a superior PFS benefit than osimertinib, although no efficacy advantage was observed [36]. In our present study, we enrolled 36 patients with uncommon EGFR mutations treated with first‐line osimertinib, and the sample size was relatively balanced among EGFR‐TKI groups. We observed that the efficacy of osimertinib was better than chemotherapy and first‐generation EGFR‐TKIs, but no better than afatinib in all patients with uncommon EGFR mutations. In the subgroup of patients with other uncommon EGFR mutations except for L861Q and G719X, the mPFS of osimertinib seemed to be longer than afatinib (12.1 vs. 10.3 months), but the difference was not significant according to the survival curves. Nevertheless, in the present study, we did not analyze the efficacy of different EGFR‐TKIs in patients with central nervous metastasis owing to the small sample size of each group. However, considering that osimertinib had higher permeability of blood–brain barrier [37], it could be a better choice for patients harboring uncommon EGFR mutations with central nervous metastasis. OS did not show a remarkable difference among these four groups in our study, for the reason that many factors affect OS outcomes including sample size and follow‐up treatments.

Safety is an important factor in influencing the quality of life and survival of patients. In accordance with previous results, the safety of osimertinib is better than first‐generation and second‐generation of EGFR‐TKIs [38, 39]. When treated with afatinib, patients should pay attention to the management of safety, especially for gastrointestinal‐related AEs and rash.

There were still some limitations in our present study. First of all is its retrospective nature. Secondly, considering the heterogeneity of uncommon EGFR mutations, different types of uncommon EGFR mutations should be analyzed individually. However, owing to the small sample size, we were unable to do so. Further studies with larger sample sizes are needed to sub‐classify different types of uncommon EGFR mutations to find a better treatment strategy.

In summary, our data reviewed that NSCLC patients with non‐ex 20 ins uncommon EGFR mutations benefited better from EGFR‐TKIs compared with chemotherapy as first‐line therapy. Afatinib performed with great efficacy for the majority of non‐ex 20 ins uncommon EGFR mutations, especially in the L861Q mutation. Osimertinib also showed efficacy in patients with non‐ex 20 ins uncommon EGFR mutations, especially in those without L861Q and G719X mutations. Safety also should be a concern when choosing EGFR‐TKI for treatment, patients should pay attention to the management of safety when using afatinib while osimertinib is safer.

## Author Contributions

**Chen Liao:** writing – original draft (equal). **Li Bai:** supervision (equal). **Tingting He:** writing – original draft (equal). **Qingle Liang:** software (equal). **Defeng Hu:** methodology (equal). **Shipeng Lei:** formal analysis (equal). **Yong He:** supervision (equal). **Yubo Wang:** resources (lead).

## Ethics Statement

This study has been approved by the Ethics Committee of Chongqing University Jiangjin Hospital [Ratification No.:KY2023024] and the informed consent waiver has been obtained. We guarantee that this study was conducted in accordance with the Declaration of Helsinki.

## Conflicts of Interest

The authors declare no conflicts of interest.

## Data Availability

The authors confirm that the data supporting the findings of this study are available within the article.
